# Three *Staphylococcus* Bacteriophages Isolated from Swine Farm Environment in Quebec, Canada, Infecting *S. chromogenes*

**DOI:** 10.3390/v18010146

**Published:** 2026-01-22

**Authors:** Mousumi Sarker Chhanda, Rébecca E. St-Laurent, Valérie E. Paquet, Nicolas Deslauriers, Cynthia Gagné-Thivierge, Martine Denicourt, Marie-Ève Lambert, Antony T. Vincent, Steve J. Charette

**Affiliations:** 1 Institut de Biologie Intégrative et des Systèmes (IBIS), Université Laval, Quebec, QC G1V 0A6, Canada; mousumi-sarker.chhanda.1@ulaval.ca (M.S.C.); rebecca.st-laurent.1@ulaval.ca (R.E.S.-L.); valerie.paquet@ibis.ulaval.ca (V.E.P.); nicolas.deslauriers.1@ulaval.ca (N.D.); cynthia.gagne-thivierge.1@ulaval.ca (C.G.-T.); antony.vincent@fsaa.ulaval.ca (A.T.V.); 2 Département de Biochimie, de Microbiologie et de Bio-Informatique, Université Laval, Quebec, QC G1V 0A6, Canada; 3 Centre de Recherche en Infectiologie Porcine et Avicole (CRIPA)—Fonds de Recherche du Québec—Nature et Technologies, Saint-Hyacinthe, QC J2S 2M2, Canada; martine.denicourt@umontreal.ca (M.D.); marie-eve.lambert@umontreal.ca (M.-È.L.); 4 Département des Sciences Animales, Faculté des Sciences de l’Agriculture et de l’Alimentation, Université Laval, Quebec, QC G1V 0A6, Canada; 5 Département de Sciences Cliniques, Faculté de Médecine Vétérinaire, Université de Montréal, Saint-Hyacinthe, QC J2S 2M2, Canada

**Keywords:** bacteriophages, *Staphylococcus*, swine, *Staphylococcus chromogenes*, exudative epidermitis (EE)

## Abstract

Exudative epidermitis (EE), caused by *Staphylococcus hyicus*, represents an issue for swine production, particularly due to antimicrobial resistance. In this project, we isolated bacteriophages using *S. hyicus* as host and studied them as a potential alternative to antibiotic treatment in Quebec, Canada. Three phages, STAE-4, STAF-3, and STAM-1, were isolated from swine farm samples using a single *S. hyicus* strain (SC366) as the host. Transmission electron microscopy revealed that all three phages exhibited a siphovirus-like morphology, and RAPD-PCR profiling indicated that the phages were genetically distinct. Whole genome sequencing confirmed these differences and showed that the three phages were closely related to each other, and, more importantly, highly similar to phages previously described as infecting *Staphylococcus chromogenes*, a species closely related to *S. hyicus*. Host range analysis confirmed that the three phages preferentially infected the *S. chromogenes* strains included in the study, exhibiting minimal to no lytic activity against other strains of *S. hyicus* or *Staphylococcus agnetis*, another closely related species. The only exception was the host *S. hyicus* strain SC366, which was effectively infected by all three phages, albeit less efficiently than the most sensitive *S. chromogenes* strain (SC385). Adsorption tests further supported these observations, showing that phages bound to strain SC366 much more quickly than to SC385, despite the lower lytic activity observed. Taken together, these results highlight that while the phages retain some capacity to infect *S. hyicus*, their biological properties point to a stronger adaptation to *S. chromogenes*, indicating that they are not suitable candidates for controlling EE.

## 1. Introduction

Bacteria from the *Staphylococcus* genus are found in humans, animals, and the natural environment. Several pathogenic species of this genus cause significant diseases in livestock, such as swine and ruminants. Among them, *Staphylococcus hyicus*, a Gram-positive facultative anaerobe, is the main causative agent of exudative epidermitis (EE), also known as greasy pig disease, a skin disease affecting young piglets [1,2,3,4]. This disease causes skin dermatitis, wasting, dehydration and can lead to a variable mortality rate [5,6]. The bacteria can also be involved in cases of arthritis. The transmission of this disease mainly occurs horizontally via skin lesions and direct contacts or vertically during the birth of the piglet [1].

Closely related to *S. hyicus* are *Staphylococcus chromogenes* and *Staphylococcus agnetis*, two species that also colonize the skin and mucosa of animals [7,8,9]. *S. agnetis* is an emerging pathogen in both poultry and cattle. *S. chromogenes* occurs frequently in cattle, pigs, and even poultry. In cattle, it is mainly associated with mastitis, whereas in pigs and poultry it is generally linked to other opportunistic infections. Although *S. chromogenes* is most likely to cause problems in cattle, it is also known to cause infections in pigs [10].

EE is a commonly diagnosed disease in suckling piglets [11]. To reduce the risk of disease, various management strategies are employed [12]. Farm facilities are also disinfected, in part with chlorhexidine-based sprays [1]. Nonetheless, antibiotic treatment remains a primary option for managing EE. Controlling the disease has become increasingly challenging due to the rising antimicrobial resistance of *S. hyicus* strains; resistance against macrolides, tetracyclines, sulfonamides, streptomycin, penicillin, ampicillin, and ceftiofur has been reported in previous studies [12,13,14,15].

Overall, the global rise of antimicrobial resistance in the treatment of microbial diseases in farm animals, including swine, has prompted researchers to explore alternative therapeutic approaches [12,13,14]. Bacteriophages (or phages), which are viruses that specifically infect bacteria, are considered to be one of the other possible alternatives for treating bacterial diseases such as EE [16]. Phages are expected to offer a specific and sustainable approach for the treatment of microbial diseases [16,17,18,19]. The skin-localized nature of EE makes it well-suited for topical phage application and the narrow host range of phages reduces the risk of disrupting the surrounding microbiota of the target environment [20].

Early research (1980s–1990s) on *S. hyicus* phages focused primarily on their usage as typing tools for epidemiological studies in Japan, Belgium, Slovakia, the Czech Republic, and Denmark from swine with or without EE. While these studies did isolate phages for typing purposes, they did not undertake detailed characterization of their biological properties or genomic features [21,22,23]. More recently, two studies have isolated and identified *S. hyicus* phages with the specific goal of developing phage-based treatments for EE in central Europe (Germany and the Czech Republic) [24,25].

For this type of therapeutic applications in swine farms, it is important to isolate and characterize a diverse set of *S. hyicus* phages using host strains from the same geographic region where the treatment is intended, as phylogenetic analyses indicate that *S. hyicus* populations can differ across regions [26]. To date, no *S. hyicus*-infecting phages have been isolated in Quebec. Therefore, the present study was designed to isolate and identify bacteriophages able to infect *S. hyicus* from Quebec. The morphology, genetic diversity, and host susceptibility of the isolated phage were analyzed to evaluate their potential use in future phage-based treatments for EE. Although the three phages were isolated using *S. hyicus* as the host, genomic and host range analyses suggest that they are more closely related to phages that infect *S. chromogenes*.

## 2. Materials and Methods

### 2.1. Bacterial Strains Used in This Study

A total of 80 *Staphylococcus* spp. strains were included in this study (Table S1), comprising 49 *S. hyicus*, 17 *S. agnetis*, and 14 *S. chromogenes* strains. The bacterial strains analyzed in this study originated from in-house clinical collections, including *S. hyicus* from a swine clinical environment and *S. chromogenes* and *S. agnetis* from a bovine clinical environment. Selection criteria included their availability, Canadian geographic origin, and close phylogenetic relationships (Figure S1). They were generously provided by the Laboratoire de santé animale (MAPAQ) in Saint-Hyacinthe (*S. hyicus*) and by the Canadian Bovine Mastitis Research Network with support from Dairy Farmers of Canada, Agriculture and Agri-Food Canada, and the Université de Montréal (*S. chromogenes* and *S. agnetis*). Genome sequences of 20 of these strains were already available at the outset of the project (Table S1).

### 2.2. Phylogenetic Analysis

A molecular phylogenetic analysis of the genus *Staphylococcus*, focusing on species closely related to *S. hyicus*, was conducted as previously described [26] to guide the optimal choice of *S. hyicus* strains for phage isolation. The analysis included 20 sequenced strains from our laboratory (Table S1), along with representative reference genomes retrieved from NCBI (Table S2). Six *S. hyicus* strains (SC302, SC304, SC310, SC366, SC386, SC390), isolated from piglets infected by *S. hyicus* in Eastern Canada, were selected to encompass as much as possible the phylogenetic diversity observed among the strains available, and served as hosts for phage isolation. (Figure S1).

### 2.3. Sample Collection

One 50 mL water sample was collected from the floor of the pens where animals had been present. Slurry samples (50 mL each) were obtained from the pen floor (*n* = 1) and from the pipe leading to the pit (*n* = 2). A 50 mL feed sample was collected directly from a trough containing feed mixed with water that had also been in contact with the animals. At the time of sampling, no cases of EE were detected in this Quebec farm, as expected in a grow-finish swine operation. For confidentiality reasons, the precise location is not disclosed.

### 2.4. Phage Isolation

For swine manure and feed samples, 5 mL of each sample were added to 40 mL of 1× phage buffer (50 mM Tris-HCl pH 7.5, 100 mM NaCl, 8 mM MgSO_4_) and the mixture was incubated in a tube rotator at room temperature for approximately 18 h. After incubation and diffusion, each sample was centrifuged three times for 10 min at 3200× *g* at room temperature, after which the supernatants were filtered into a new sterile tube using a 50 mL syringe with 0.45 µm filters. Water samples were centrifuged and filtered once.

*S. hyicus* strains selected as hosts for phage isolation were grown from freezer stocks on Tryptic Soy Agar (TSA, 800,055 CG, Wisent Inc., Saint-Jean-Baptiste, QC, Canada) at 37 °C for 24 h before being used in subsequent experiments.

For each trial of the phage amplification, 5 mL of 2× concentrated Tryptic Soy Broth (TSB, 800,056 CG, Wisent Inc., Saint-Jean-Baptiste, QC, Canada) was mixed with a 5 mL sample and 1% *v*/*v* of a bacterial culture in exponential growth phase in a sterile tube. The tube was incubated at 37 °C for 24 h in agitation at 150 rpm. Following incubation, cultures were centrifuged at 3200× *g* for 10 min and the supernatants were filtered through a 0.45 µm syringe-mounted filter to remove bacterial cells. To detect the presence of phages, spot tests were performed. A 10 µL drop of each filtered lysate was dropped onto a bacterial lawn prepared using the corresponding isolation strain. The lawn was prepared by mixing 100 µL of the bacterial culture in 3 mL of warm soft agar (TSB supplemented with 0.75% agar, maintained at 55 °C) then poured onto thin TSA plates. Bacterial lysis indicated the presence of phages in the sample. To obtain a more concentrated phage preparation, a second round of amplification was conducted under the same condition using 5 mL of TSB (1×) and 5 mL of the lysate from amplification. Phage-positive lysates were subsequently stored at 4 °C until further experimentation.

The amplified phages were purified through multiple rounds of titration. Initially, phage lysates were serially diluted up to 10^−5^ in 1× phage buffer (900 µL). For each dilution, 100 µL of phage lysate dilutions and 100 µL of host strain culture were mixed with 3 mL of warm soft agar and poured onto thin TSA plates. Plates were incubated at 37 °C for 24 h. Following incubation, a truncated pipette tip was used to collect five well-isolated lysis plaques from each positive sample. Then, the plaques were transferred into separate glass tubes containing 500 µL of 1× phage buffer for at least 30 min of diffusion. A second round of isolation was then performed for each clone (in duplicate) using dilutions up to 10^−2^. In the third round, a single isolated lysis plaque was picked per clone and transferred into 500 µL of 1× phage buffer for final diffusion. After that, the final amplification was conducted with the total volume of isolated phage diffusion (500 µL), 10 mL TSB 1× and 100 µL of the bacterial host strain. After 24 h of incubation at 37 °C and 150 rpm, the phage amplification was centrifuged and filtered to obtain purified phage lysate. These purified phage lysates were stored at 4 °C for subsequent analysis.

Titration was also performed to determine the concentration of phage lysates prior to long-term storage. Phage lysates were serially diluted up to 10^−7^ dilution in 1× phage buffer. For the 10^−4^ to 10^−7^ dilutions, 100 µL of phage suspension were mixed with 100 µL of an exponentially growing bacterial culture and 3 mL of warm soft agar, then poured onto thin TSA plates. The plates were incubated at 37 °C for 24 h. After incubation, lysis plaques were counted to determine the phage concentration (titer). Plates with 30 to 300 plaque-forming units (PFU) were used to calculate the phage titer using the following formula:

$$Phage\;titer\;(PFU/mL)=\frac{Number\;of\;PFU}{Volume\;(mL)}\times \frac{1}{Dilution\;factor}$$

High-titer phage clones were selected for long-term storage. If the titer values were not high (i.e., <10^8^), then the plate lysate method [27] was used to increase the phage titer of each clone. For each clone, 4 to 6 identical Petri dishes were used as described for titrations (see previous paragraph), using the tube containing the isolated lysis plaque. After incubation, this resulted in a bacterial lawn with near-complete lysis. Subsequently, 5 mL of 1× phage buffer was added to the surface of the two bacterial lawns. A cell scraper was used to mix the soft agar into the buffer and permit the phage diffusion. The soft agar and buffer mixture from both dishes was pooled into a single tube. The tube was gently rotated at room temperature for 30 min to continue the phage diffusion, then centrifuged at 3200× *g* for 10 min. The supernatant was filtered using a 10 mL syringe and a 0.45 µm filter. These steps were repeated with the remaining Petri dishes, using the previously obtained filtrate instead of fresh phage buffer to gradually concentrated the 1× phage buffer. The resulting phage lysate was titrated and stored at 4 °C. To preserve them on the long term, 850 µL of phage lysate were mixed with 150 µL of sterile glycerol in cryotubes. The tubes were immediately placed on dry ice after mixing and then stored at −70 °C.

### 2.5. Transmission Electron Microscopy

The morphology of isolated phages was examined using transmission electron microscopy (TEM). For sample preparation, 8 mL of each phage lysate were transferred to a clean 10.4 mL Beckman coulter tube (355603, Beckman Instruments Inc., Mississauga, Ontario Canada) for ultracentrifugation at 25,000× *g* for 1 h at 4 °C. Following centrifugation, the supernatant was carefully removed, leaving approximately 500 µL in each tube to avoid disturbing the phage pellet. To wash the phage pellet, 5 mL of 0.1 M ammonium acetate were added, and the tubes were centrifuged again under the same conditions. The washing step was repeated twice. After the final centrifugation, approximately 200 µL of the concentrated phage suspensions were collected into 1.5 mL microcentrifuge tubes and stored at 4 °C until TEM observation.

The phage preparation was used to prepare observation grids for electron microscopy. Negative staining was performed using 2% uranyl acetate (30 s) applied to copper grids. The prepared grids were observed with a JEOL 2100 Plus transmission electron microscope (JEM 2100Plus, JEOL Ltd., Saint-Hubert, QC, Canada), at the microscopy platform of the Institut de Biologie Intégrative et des Systèmes (Université Laval). All images were acquired at a voltage of 200 kV and a magnification of 50,000× to ensure consistency across samples. Head and tail dimensions were measured in nanometers (nm) using ImageJ software (version 2.16.0/1.54p) running on Java 1.8.0_322 (64-bit) [28]. Measurements were taken from 10 images per sample to calculate average dimensions.

### 2.6. Randomly Amplified Polymorphic DNA- PCR

Randomly Amplified Polymorphic DNA (RAPD) PCR was performed on the isolated phages to have an initial assessment of their genetic diversity. Phage DNA was extracted from the lysates using the Norgen^®^ Phage DNA Isolation Kit (46850, Norgen Biotek corp, Thorold, ON, Canada), according to the manufacturer’s protocol. Lysate of the SC366 (0.5 µL) strain was used as a control. The lysate was prepared as previously described [29]. PCR amplification was carried out using GoTaq^®^ DNA polymerase (PR M3008, Promega Corporation, Fitchburg, WI, USA) and the RAPD #18 primer (5′-GCCAGCAGG-3′) [30]. The thermal cycling conditions consisted of denaturation at 95 °C for 45 s, annealing at 37 °C for 45 s, and extension at 72 °C for 2 min and 30 s. This cycle was repeated 40 times. PCR products were separated by electrophoresis on a 1% agarose gel and ran for 70 min at 100 V. Following electrophoresis, the gel was stained with weak agitation with ethidium bromide (0.5 μg/mL) for 30 min, then destained in a water bath with weak agitation for 1 h before visualization under UV light [31].

### 2.7. DNA-Extraction for Genome Sequencing

Phage DNA was extracted for genome sequencing using the DNeasy blood and tissue kit (Qiagen, Montreal, QC, Canada) with modifications has indicated previously [30]. Purified DNA samples were then submitted to Plasmidsaurus (Eugene, OR, USA) for whole genome sequencing using the Illumina NextSeq platform, which produces paired-end short reads.

### 2.8. Genomic Analysis

#### 2.8.1. Phage Genome Assembly and Protein Annotation

Raw reads were filtered using fastp v0.23.4 [32] with default parameters and filtered reads were de novo assembled into genomes using Shovill [33] v1.1.0 with default parameters. Protein annotation was performed using Pharokka [34] v1.7.5 and phold v0.2.0 [35] with default parameters.

#### 2.8.2. Genomic Comparison

The genomes of three phages (STAE-4, STAF-3, and STAM-1) isolated in this study were compared with seven phages described in recent studies and known to infect *S. hyicus* and related species [24,36,37]: phage Pel11 (GenBank accession: PP971698.1), phage Pel53 (GenBank accession: PP952070.1), phage PMBT8 (GenBank accession: MK893987.1), phage PMBT9 (GenBank accession: MW221967.1), phage PT94 (GenBank accession: PP212903.1), phage PT1-1 (GenBank accession: PP212900.1), and phage PT1-4 (GenBank accession: PP212901.1). The Percentage of Conserved Proteins (POCP) between the proteomes of all phages was calculated using pocp v2.3.6 [38,39] and a pairwise matrix was constructed. The matrix was then transferred to an R environment using R v4.4.1, and a hierarchical cluster analysis was performed. Briefly, the pvclust v2.2.0 R package [40] was used with the average clustering method, an Euclidean-based dissimilarity matrix, and a multiscale bootstrap value of 1000 replicates. The cluster dendrogram obtained was extracted as a newick file and visualized into iTOL v7 [41]. To identify genome differences, tBLASTx comparisons between all phage genomes were performed and visualized using Easyfig v2.2.5 [42]. The values of average nucleotide identities (ANIs) were calculated using pyANI-plus version 0.0.1.

#### 2.8.3. Identification of Antibiotic Resistance Genes and Virulence Factors

All phage genomes were screened for the presence of antibiotic resistance genes and virulence factors as part of Pharokka’s pipeline. Briefly, all coding sequences were matched to the Comprehensive Antibiotic Resistance Database [43] and the Virulence Factor Database [44] using MMseqs2 [45] and PyHMMER [46].

### 2.9. Host Range and Efficiency of Plating

A total of 80 bacterial strains were used to assess the host range of the isolated phages, including 49 *S. hyicus*, 17 *S. agnetis*, and 14 *S. chromogenes* strains (Table S1). Phage lysates were standardized to a concentration of 10^7^ PFU/mL, and each assay was duplicated to ensure reproducibility. For each test, 100 µL of an exponentially growing bacterial culture was mixed with 3 mL of warm soft agar and immediately poured onto thin TSA plates to form a uniform bacterial lawn. Phage samples were serially diluted in phage buffer up to 10^−7^. A 10 µL drop from each dilution was spotted onto the bacterial lawn. After drying, the plates were incubated at 37 °C for 24 h, and lysis plaques were observed. Strain susceptibility was categorized based on the dilution at which the lysis was observed. The efficiency of plating (EOP) values were calculated from the host range experiment and were obtained by comparing results from the reference host strain SC385 and the other sensitive strains. For each tested strain, the value of the tested strain has been divided by the value of the host strain (optimal lytic efficiency) and multiplied by 100 to obtain the EOP.

### 2.10. Phage Stability and One-Step Growth Curve

To assess phage stability, we selected STAF3 because the other phages analyzed in this study are closely related to STAF3 and are therefore expected to exhibit comparable stability profiles. For the stability assay, an initial titer of 1.8 × 10^7^ PFU/mL was used. Phage STAF3 was incubated under three temperature conditions: 4 °C (recommended for long-term phage storage), room temperature (21–23 °C), and 31 °C (a temperature that mimics the head skin surface temperature of pigs as measured by a veterinarian). The objective was to assess the suitability of STAF3 under different conditions. Phage titers were quantified at defined intervals using standard plaque assays.

A one-step growth curve assay of STAF3 on its host strain *S. chromogenes* SC385 was performed three times as reported previously [47] with modifications. Approximately 10^9^ CFU of cells per mL (1 mL of a culture in exponential phase with an optical density between 0.5 and 0.55) were harvested by centrifugation and resuspended in 900 μL of TSB. Phages were added at a multiplicity of infection (MOI) of 0.1 and allowed to adsorb for 10 min at 37 °C. Then, infected cells were harvested by centrifugation, and the pellet was washed twice with 1 mL of fresh TSB. The pellet that contained the infected cells was resuspended in a final dilution of 1:1000 in 100 mL of fresh TSB in a sterile Erlenmeyer flask. Before being incubated at 37 °C at 150 rpm, the initial titer was sampled in technical triplicate on a soft top agar plate. Every 10 min, an aliquot of 500 μL was centrifuged to remove phages that did not adsorb. The supernatant was plated in technical triplicate to determine the phage titer, up to 90 min. The burst size and the burst time were calculated as already reported [47]. The size of 40 random lytic plaques was measured after 24 h of incubation on the same plates used for the one-step growth curve assay.

### 2.11. Phage Adsorption

Two bacterial strains (*S. hyicus* SC366 and *S. chromogenes* SC385) were considered for the adsorption test based on their high sensitivity to the phages. Bacterial cultures were used that had been grown in TSB at 37 °C with agitation to an optical density at 600 nm between 0.6 and 0.8. Phage stocks were freshly diluted to a concentration of 1.5 × 10^4^ PFU/mL in 100 µL immediately before each experiment. For each trial, 100 µL of the diluted phage solution were mixed with 900 µL of TSB (control) or fresh bacterial suspension (adsorption conditions), and the mixture was incubated at 37 °C using a tube rotator at 150 rpm for 10 or 20 min. Following incubation, the samples were centrifuged at 17,000× *g* for one minute to pellet the bacteria. Supernatants were carefully collected and mixed with fresh host bacteria, then plated using the soft agar overlay method [48,49]. After overnight incubation at 37 °C, lytic plaques were counted. The final adsorption efficiency was calculated using the following formula [50]:

$$Adsorption(\%)=\frac{{Titer}_{control}-{Titer}_{adsorped}}{{Titer}_{control}}\;\times 100$$

To ensure statistical robustness, each condition was performed in triplicate [48,49], and the results were averaged for analysis. Data analysis was conducted by using the tidyverse package [51] in R (v 4.3.0) [52]. The mean and standard deviations of adsorption rates were calculated for each combination of three phage, two bacterial strains, and two time points. Line plots with error bars were generated to illustrate phage adsorption dynamics over time for the bacterial strains SC366 and SC385.

## 3. Results

### 3.1. Phage Isolation

Out of six *S. hyicus* strains used for phage isolation in this study, with all the environmental samples used, only strain SC366 yielded positive results. Three distinct phages were isolated from water, manure, and feed mixed with water samples using this bacterial strain. Lysis was observed for both liquid and solid media methods (Table 1). Following the first round of amplification, three samples produced clear lysis zones on solid media, enabling subsequent phage isolation. Phages recovered from solid media generated liquid lysis during the second amplification step. From each of the three samples, one phage clone was isolated and purified, resulting in three distinctive phages named vB_SchS_STAE-4, vB_SchS_STAF-3 and vB_SchS_STAM-1, referred to hereafter as STAE-4, STAF-3, and STAM-1 for simplicity. These clones were selected based on their ability to produce extensive lysis on agar. The lysis plaques produced by each phage were clear and measured less than 1 mm in diameter. After titration, all three phages displayed titers of at least 10^8^ PFU/mL were subsequently stored at −70 °C for long-term storage.

### 3.2. Phage Morphology

**Figure 1:**
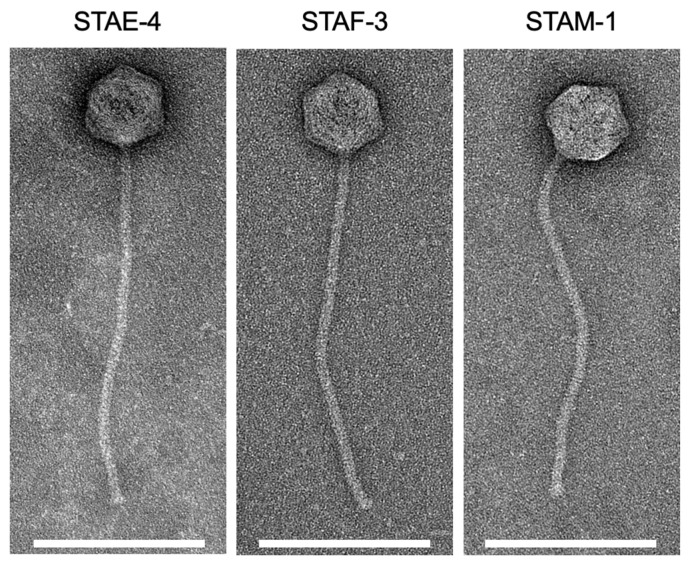
Transmission Electron Microscopy Images of Bacteriophages STAE-4, STAF-3, and STAM-1, at 200 kV and 50,000× magnification (white scale bar: 200 nm).

The morphology of phages STAE-4, STAF-3, and STAM-1 was examined by TEM (Figure 1). All three phages have almost the same morphological features, characterized by an isometric capsid and a long, non-contractile tail. In all cases, the tail was approximately 4.2 to 4.7 times longer than the capsid diameter (Table 2). Based on these morphological traits, all three phages have a siphovirus-like morphology [53,54].

### 3.3. RAPD-PCR

**Figure 2:**
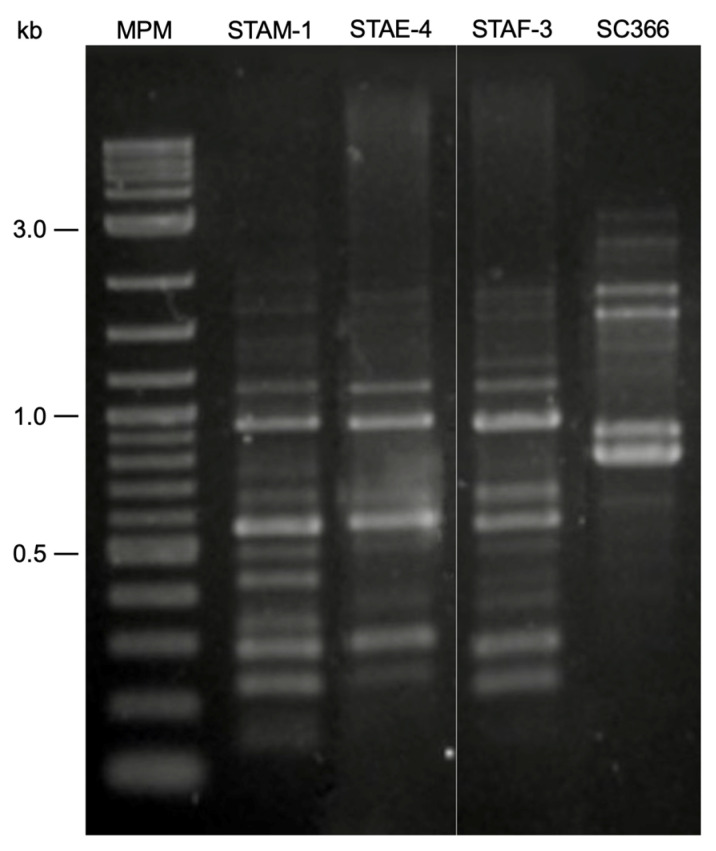
RAPD-PCR profiles of the isolated phages STAM-1, STAE-4, and STAF-3. The band on the left corresponds to the molecular weight marker (MPM; Quick-Load^®^ 1 kb Plus DNA Ladder, New England Biolabs, Ipswich, MA, USA). DNA lysate from the host strain SC366 was included as a control. The white line indicates that the image is a composite, showing non-adjacent sections of the original gel.

To investigate the genetic differences among the isolated phages, RAPD-PCR analysis was performed (Figure 2). The results showed that the three phages share some banding patterns, indicating possible regions of genetic similarity. However, each phage also exhibited distinct bands and intensity profiles, especially for bands below 500 bp, that differentiated them from each other. STAM-1 showed a greater number of prominent bands, suggesting a potential higher genetic divergence than the other phages.

### 3.4. Genomic Analysis

**Figure 3:**
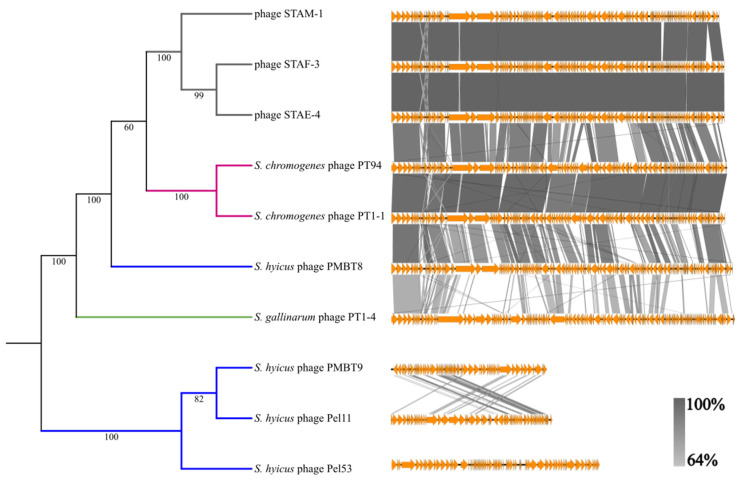
A comparison of the phage genomes analysed in this study. A Cluster dendrogram of the phage genomes based on the percentage of conserved protein matrix is shown on the left. The colors of the branches represent the host species of phages: *Staphylococcus hyicus* (blue), *Staphylococcus chromogenes* (pink), and *Staphylococcus gallinarum* (green). Bootstrap values are indicated for each branch. tBLASTx comparisons of the phage genomes visualized with Easyfig are shown on the right. Each genome is represented on a different line according to their position on the cluster dendrogram. Orange arrows represent coding sequences. Vertical blocks between genomes indicate regions of shared similarity according to the tBLASTx analysis and are colored based on percentage of similarity: from low similarity (light grey) to high similarity (dark grey).

Table S3 summarizes the main genomic features of the three phages isolated in this study. These phages exhibit similar characteristics, including a GC content of 31.6% and genome sizes ranging from 84.6 to 85.9 kb. Approximately 35% of the predicted genes could be assigned a known or putative function. Although these phages were isolated using an *S. hyicus* strain, they are more closely related to those of *S. chromogenes*-infecting phages PT94 and PT1-1 than to other known *S. hyicus* phages in GenBank based on the phylogeny analysis. *S. hyicus*-infecting phages are not confined to a single phylogenetic clade but are instead distributed across distinct lineages. Among them, only phage PMBT8 shares both a genome length similar to that of STAE-4, STAF-3, and STAM-1, and a high level of sequence identity (Figure 3).

Phages STAE-4, STAF-3, STAM-1, PT94, PT1-1, PMBT8, and PT1-4 can be considered part of a same family based on their sequence similarity and genome size (Figure 3). A heatmap of pairwise comparisons of conserved protein content (Figure S2) further supports this grouping, with PT94 and PT1-1 showing the highest similarity to the three phages characterized in this study, followed by PMBT8. In contrast, PT1-4 exhibits lower similarity to all other members of the group. In contrast, the average nucleotide identity (ANI) between the three phages isolated in this study and their closest known relatives (Table S4) in combination with the percentage of coverage between them (Table S5) identifies PMBT8 as the closest relative of STAE-4, STAF-3, and STAM-1.

The three newly isolated phages (STAE-4, STAF-3, STAM-1) exhibited highly similar genomic organization, each encoding 157–159 CDS compared to 151 in PT1-1 and PT94 (Table S6). Most genes were of unknown function (111 vs. 102–105 in PT1-1/PT94). Among annotated categories, DNA/RNA metabolism genes were most abundant (22–24 CDS), followed by structural modules for head (6–7 CDS) and tail (4 CDS). Lysis genes were slightly more numerous in the new isolates (3 vs. 2), and integration/excision genes were present only in these phages, suggesting temperate potential. Overall, the new phages share a conserved architecture with previously described phages but differ in CDS count and integration-related genes. We did not detect any antibiotic resistance genes or virulence factors within the phages isolated in this study.

### 3.5. Host Range and Efficiency of Plating

To assess the host range of the three isolated phages, a total of 80 bacterial strains available in our collection were tested, including 49 *S. hyicus*, 17 *S. agnetis*, and 14 *S. chromogenes* strains (Table 3). Only a small number of strains were susceptible to the phages. The most striking result is that all three phages exhibited their lytic activity mainly against *S. chromogenes* strains included in the study. More specifically, 5 of the *S. chromogenes* strains demonstrated either intermediate or high sensitivity to all three phages, with strains SC385 and SC437 being the most sensitive. Overall, 36% of the *S. chromogenes* strains tested showed at least intermediate susceptibility to the phages.

Outside of the *S. chromogenes* strains that were targeted by all three phages, only one *S. hyicus* strain displayed intermediate sensitivity to them, while three other *S. hyicus* strains showed low-level susceptibility to one, two, or all three phages. Notably, the most susceptible *S. hyicus* strain was SC366, the same strain used as the host for phage isolation from farm samples. Phage STAM-1 was also able to infect one *S. agnetis* strain (SC298, intermediate susceptibility), which showed low-level sensitivity to the other two phages as well. Another *S. agnetis* strain (SC355) was also weakly sensitive to STAM-1. However, neither *S. hyicus* nor *S. agnetis* strains exhibited the consistent susceptibility to all three phages that characterized a significant fraction of the *S. chromogenes* strains. This observation is consistent with the phylogenomic analysis and the percentage of conserved proteins, which indicate that although these phages were isolated using *S. hyicus* strains, they are most similar to previously described phages that infect *S. chromogenes*. The EOP values for each phage are presented in the same table as the host-range results (Table 3), facilitating direct comparison of lytic activity and plating efficiency.

### 3.6. Phage Stability and One-Step Growth Curve

Because STAF3 clustered closely with the two other newly isolated phages in the phylogenetic tree, we selected STAF3 for more in-depth characterization of this phage group. STAF3 remained stable for 6 months at 4 °C without significant loss of titer. At room temperature, the phage maintained its original titer for 8 days, while at 31 °C, stability was observed for 2 days. After prolonged exposure at 31 °C, titers decreased to 1.50 × 10^4^ PFU/mL after 4 days and 1 × 10^2^ PFU/mL after 8 days, indicating a marked loss of infectivity at elevated temperatures.

**Figure 4:**
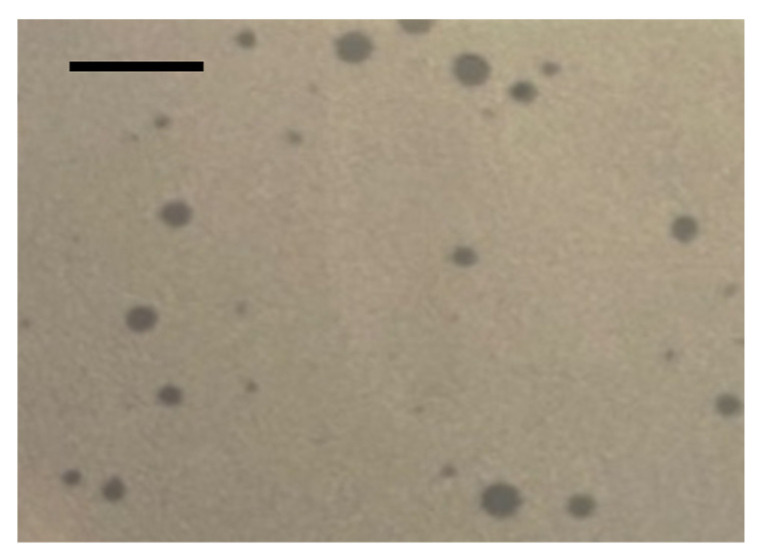
Morphology of lytic plaques of STAF3 grown on *S. chromogenes* SC385 strain after 24 h of incubation at 37 °C on TSA medium. Scale bar = 5 mm.

**Figure 5:**
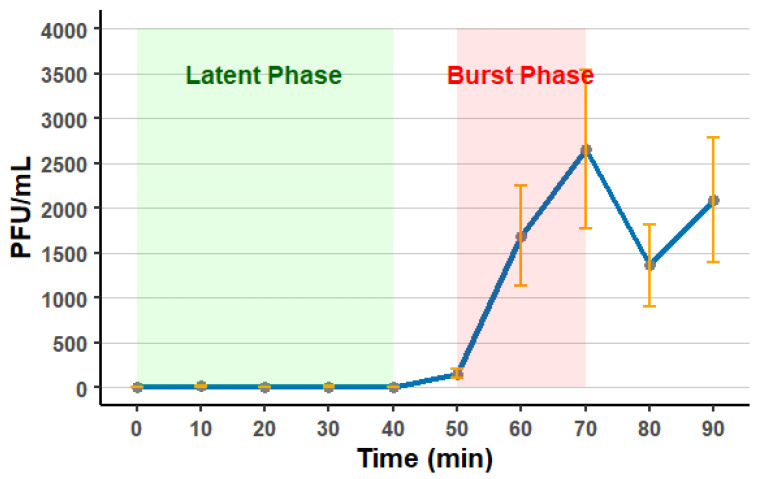
One-step growth curve of STAF3. The burst size of STAF3 was 19 ± 5 PFU per cell, with a lysis time of 72 ± 3 min. The curves represent three independent biological replicates performed using the host *S. chromogenes* SC385, with enumeration in technical triplicate for each experiment. Error bars represent the standard error of the mean.

STAF3 produced clear lytic plaques with an average diameter of 0.97 ± 0.33 mm (Figure 4) and plaque size appeared variable. Its burst size when infecting SC385 was estimated at 19 ± 5 PFU per cell, with a lytic cycle duration of 72 ± 3 min (Figure 5).

### 3.7. Adsorption

**Figure 6:**
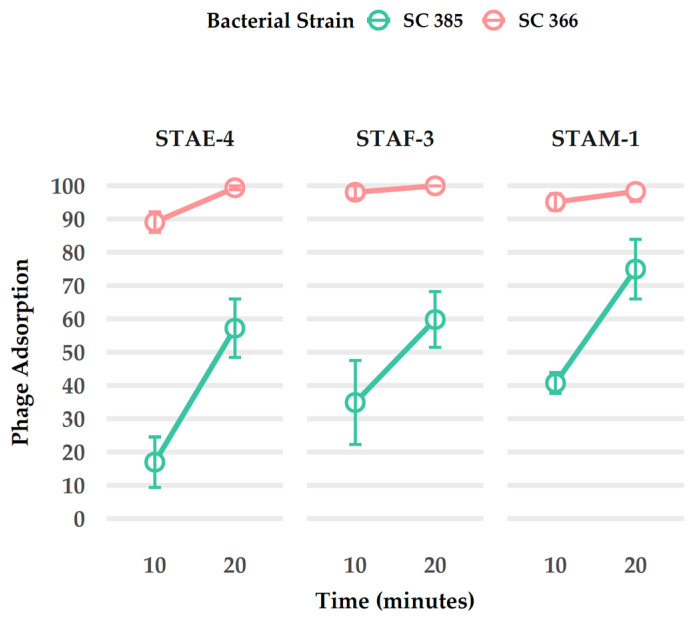
Adsorption dynamics of phages STAE-4, STAF-3, and STAM-1 on two bacterial strains, SC385 and SC366, at 10 and 20 min. Adsorption efficiency was calculated by comparing the number of lysis plaques before and after adsorption for each time point using TSA top agar plates. Each experiment was conducted with a minimum of three biological replicates, each including three technical replicates.

Phage adsorption assays revealed strain-dependent variations in adsorption efficiency. The *S. hyicus* SC366 strain exhibited higher adsorption rates for phages STAE-4, STAF-3, and STAM-1 compared to the *S. chromogenes* strain SC385 at both 10 and 20 min (Figure 6). Overall, the results indicate that all three phages rapidly bind to SC366, and that both bacterial strains show a general increase in phage adsorption over time.

## 4. Discussion

### 4.1. Phage Isolation, Morphology and Stability

This study was designed to isolate phages and test their efficacy for the potential treatment of piglets infected with *S. hyicus.* Three phages, STAE-4, STAF-3, and STAM-1, were successfully isolated from water, manure from the pit, and feed mixed with water samples from one swine farm in Quebec, Canada where EE was not present. While numerous studies have focused on the isolation and therapeutic application of bacteriophages, particularly those that target *S. aureus*, research on phages specific to *S. hyicus*, the causative agent of EE, remains limited [37,55,56,57,58]. To date, only a few studies have reported the isolation of phages active against *S. hyicus*, and among these, only two have been confirmed as strictly virulent. In addition, three *S. chromogenes* phages were also isolated from dairy farm sewage; however, no *S. chromogenes* phages have been reported from swine farming environments to date [36].

Previous studies have primarily sourced phages from swine skin, feces, and eye swabs [24,25]. In this study, we used samples from less common sources for phage isolation, such as food and water found in swine enclosures. Although the isolated phages did not exhibit strong activity against *S. hyicus*, these types of sources may still be worth exploring in future phage isolation efforts. Naturally, sampling from farms with confirmed *S. hyicus* presence should increase the likelihood of isolating phages specific to this bacterium.

Phages STAE-4, STAF-3 and STAM-1 were morphologically similar, each displaying an icosahedral shape of the head and a long tail (Figure 1). All three phages exhibited a siphovirus-like morphology [53,54]. Similarly, the four phages previously described as infecting *S. hyicus* also showed siphovirus-like features [24,25]. Of the three phages isolated using *S. chromogenes* as the host, two (PT1-1 and PT94) displayed also siphovirus-like morphology, while the third one (PT1-9) displayed a podovirus-like morphology [36].

To have more clear morphological details and replication dynamics, the present study conducted the burst size or latent period for the isolated phages (Figure 5). To our knowledge, no study characterizing phages targeting *S. hyicus* or *S. chromogenes* has reported burst size or latent period. In comparison, a recent study on *S. aureus* bacteriophages showed that three viruses had latent periods between 30 and 50 min and burst sizes of 68, 220 and 280 PFU/cell [59].

Other researchers have employed RAPD-PCR profiling to identify *S. hyicus* phages from swine farms [25]. By using the same approach, it was possible to show that the three isolated phages were unique, even when they were quite similar to each other. This suggested a certain level of genetic diversity of the three isolated phages even if they were isolated from the same farm. Phages STAE-4 and STAF-3 showed more similar bands in their RAPD-PCR profile. In contrast, STAM-1 had a higher number of different bands than the previous two phages. The fact that isolated phages do not form a single clade in genomic analyses also suggests significant diversity.

Phage stability under different storage conditions is critical for therapeutic applications. STAF3 demonstrated excellent stability at 4 °C for six months, consistent with previous recommendations for phage preservation [60,61]. This suggests that STAF3 can be stored effectively for long-term use without significant loss of infectivity. At room temperature, STAF3 retained its original titer for eight days. However, at 31 °C, STAF3 exhibited rapid decrease in infectivity after two days, with titer dropping to 1.50 × 10^4^ PFU/mL at day 4 and 1 × 10^2^ PFU/mL at day 8. This means that if phages are used in environments with temperatures around 31 °C (such as on pig skin), they will remain active for approximately two days. Overall, STAF3 shows promising stability under recommended storage conditions and reasonable short-term stability at room temperature, supporting its potential for transport and field use.

### 4.2. Phage Infects S. chromogenes, Not S. hyicus

Although our initial aim was to isolate phages that target *S. hyicus*, multiple lines of evidence indicate that the three phages isolated in this study (STAE-4, STAF-3, and STAM-1) are in fact more specific to *S. chromogenes*.

Host range analysis performed using 80 *Staphylococcus* strains showed that all three phages had a narrow host range (Table 3). Most strains were either resistant or showed only low sensitivity. However, all three phages exhibited intermediate to high infectivity against various *S. chromogenes* strains. This high activity, combined with their similarity to known *S. chromogenes* phages, supports the conclusion that STAE-4, STAF-3, and STAM-1 likely target *S. chromogenes*, rather than *S. hyicus*. Note, as complementary information, that SC385 was originally misidentified as *S. hyicus*, but core genome phylogenetic analysis later confirmed its correct taxonomic assignment [62] (Figure S1).

Three phages isolated in China and capable of infecting *S. chromogenes* have been described, with their genomes available in public databases: PT1–1, PT94, and PT1–9 [36]. Among them, PT1–1 and PT94 were able to infect 80% and 93% of the *S. chromogenes* strains tested, respectively, while showing no infectivity toward strains from eight other *Staphylococcus* species examined in the same study. In contrast, PT1–9 exhibited a much narrower host range [36]. Here, STAE-4, STAF-3, and STAM-1 exhibited highly similar lysis profiles, sharing the same host range for 5 of the 14 *S. chromogenes* strains tested, corresponding to 36% of the strains. An interesting observation is that the most sensitive strain, SC385, originated from a swine environment, whereas all other *S. chromogenes* strains in this study were of bovine origin. This suggests that expanding the collection to include more porcine-derived *S. chromogenes* strains in the future could reveal a broader activity of the three phages against isolates from swine.

Based on our results, STAE-4, STAF-3, and STAM-1, are likely phages that infect *S. chromogenes*. This explains the full names we are giving to them: vB_SchS_STAE-4, vB_SchS_STAF-3 and vB_SchS_STAM-1 where SchS refers to *S. chromogenes* siphovirus.

The species *S. chromogenes* is of veterinary importance, as it is known to cause problems in cattle [62,63,64], much like *S. agnetis* [65]. To a lesser extent, it has also been reported in pigs, where it could potentially cause infections similar to EE in rare cases [1,10]. The observation aligns with its evolutionary position, as *S. chromogenes* is closely related to *S. hyicus*, the primary agent of EE in swine, having once been classified as a subspecies of *S. hyicus* and still clustering near it in multilocus phylogenetic analyses [7,66,67]. isolation of phages targeting *S. chromogenes* from environmental samples collected on swine farms highlights the relevance of this species in that context.

Protein annotation of the phage genomes revealed the presence of integrase genes in STAE-4, STAF-3, and STAM-1, suggesting their ability to integrate into the bacterial genome as prophages (Table S6). While phage-mediated gene exchange (transduction) plays a critical role in bacterial evolution [68], none of the isolated phages carry known antibiotic resistance or virulence genes, and thus pose a minimal risk of contributing to the horizontal spread of such elements. Thus, further tests will be required in the future to assess, among other aspects, whether they are truly capable of readily integrating into the bacterial genome.

### 4.3. The Uniqueness of S. Hyicus Strain SC366

To understand the reason for the higher sensitivity of the SC385 strain compared to the SC366 strain during the host range studies, we conducted an adsorption test. The results indicated a higher absorption rate in the isolated host SC366 strain for all the phages (STAE-4, STAF-3 and STAM-1) for both time points at 10 and 20 min. That might be because the phages have a strong and specific receptor or binding site to this strain even if it is not its original host [69,70,71]. Strain SC366 appears to be unique compared to other *S. hyicus* strains included in this study. This strain may be useful in future laboratory phage propagation and enrichment research.

Despite a lower adsorption capacity compared to strain SC366 during host range testing, SC385 strain presented a high susceptibility to all three phage types, producing greater lysis plaques than SC366 (Table 2). It is interesting to note that the binding of phages to SC385 was slower than SC366. This apparent difference suggests that the initial attachment of SC385 could take longer, but later stages of infection and lysis are likely highly effective.

## 5. Conclusions

In this study, we isolated three bacteriophages from non-traditional sources targeting bacteria associated with animal skin. Host range testing revealed that these phages infected only one of the 49 *S. hyicus* strains tested and exhibited the strongest lytic activity against *S. chromogenes*. Furthermore, their genomic sequences closely resemble those of previously characterized *S. chromogenes* phages, indicating that these isolates are likely specific to this species. Consequently, they are not suitable candidates for addressing EE caused by *S. hyicus*. Although the isolated phages display lytic activity against *S. chromogenes*, their potential temperate nature requires further investigation.

## Figures and Tables

**Table 1:** Summary of phage isolation from swine farm environmental sources using both liquid and solid methods. A plus sign (+) indicates lysis plaques, while a minus sign (−) indicates their absence. All assays were performed using SC366 as the host.

Phage	Source	First Amplification		Second Amplification		Titer (PFU/mL)
		Liquid Lysis	Solid Lysis	Liquid Lysis	Solid Lysis	
STAE-4	Water	−	+	+	+	1 × 10^8^
STAF-3	Manure from pit	−	+	+	+	3 × 10^9^
STAM-1	Feed + water	−	+	+	+	3 × 10^9^

**Table 2:** Average dimensions of phages observed in this study. Values represent the mean measurements (in nanometers) of 10 individual virions per phage.

Phage	Capsid Diameter (nm)	Tail Length (nm)
STAE-4	76.8 ± 2.3	355.6 ± 7.4
STAF-3	74.5 ± 2.5	330.4 ± 34.8
STAM-1	75.5 ± 5.6	338.9 ± 17.8

**Table 3:** Lytic spectrum of the three isolated phages against *S. hyicus*, *S. agnetis*, and *S. chromogenes* strains, using a standardized titer of 10^7^ PFU/mL. Strain susceptibility was categorized based on the highest dilution at which lysis was observed: resistant strains (red boxes): no lysis observed; intermediate resistant strains (orange boxes): lysis at undiluted or 10^−1^ dilution; low sensitivity (yellow boxes): lysis observed at 10^−2^; intermediate sensitivity (light green boxes): lysis at 10^−3^; high sensitivity (blue boxes): lysis observed at dilutions between 10^−4^ and 10^−7^. EOP values range from 0 (resistant), with intermediate values (0.01, 0.001, 0.5), to 1 (highly sensitive).

Strain ID	Number of Strains	Phage		
		STAE-4	STAF-3	STAM-1
*S. hyicus*	49			
SC304, SC305, SC309, SC358, SC361, SC363, SC369, SC370, SC371, SC372, SC375, SC378, SC381	13	0	0	0
SC306, SC359, SC364	3	0	0	0.001
SC367, SC388, SC393	3	0	0.001	0
SC307, SC389	2	0.001	0	0.001
SC386	1	0	0.001	0.001
SC392	1	0.001	0.001	0
SC299, SC300, SC302, SC303, SC308, SC310, SC311, SC357, SC362, SC365, SC373, SC374, SC376, SC377, SC379, SC380, SC383, SC384, SC387, SC390, SC391, SC394	21	0.001	0.001	0.001
SC368	1	0.001	0.01	0.001
SC360	1	0.001	0.01	0.01
SC382	1	0.01	0.01	0.01
SC366	1	0.5	0.5	0.5
*S. agnetis*	17			
SC342, SC347	2	0	0.001	0.001
SC341, SC343, SC344, SC345, SC346, SC348, SC349, SC350, SC351, SC352, SC353, SC354, SC356	13	0.001	0.001	0.001
SC355	1	0.001	0.001	0.01
SC298	1	0.01	0.01	0.5
*S. chromogenes*	14			
SC436, SC428, SC431, SC435	4	0	0	0
SC426, SC429, SC430, SC433, SC434	5	0.001	0.001	0.001
SC432	1	0.01	0.5	1
SC427	1	0.5	0.5	0.5
SC438	1	0.5		0.5
SC437, SC385	2	1	1	1

## Data Availability

For the present study, the three newly isolated bacteriophages genome sequences were deposited in GenBank with the following accession numbers: STAM-1 (PV929971), STAE-4 (PV929972), and STAF-3 (PV929973). The accession numbers for the bacterial genome sequences used in this study are indicated in Tables S1 and S2.

## Supplementary Materials

The following supporting information can be downloaded at: https://www.mdpi.com/article/10.3390/v18010146/s1, Figure S1. Phylogenetic tree of strains of the genus *Staphylococcus* focusing on taxon near *S. hyicus*, used as a reference to select host strains for environmental phage isolation. Figure S2. Heatmap representing the pairwise percentage of conserved proteins between all phages in this study. Table S1. Bacterial strains used in this study [72,73,74]. Table S2. Bacterial genomes used for the phylogenetic analysis presented in Figure S1. Table S3. Genomic characteristics of the three phages isolated in this study. Table S4. Average nucleotide identity (ANI) between the three phages isolated in this study and their closest known relatives. Table S5. Percentage of coverage between the three phages isolated in this study and their closest known relatives. Table S6. Number and functions of the coding sequences identified in the three phages isolated in this study and the previously described *S. chromogenes* phages PT1-1 and PT94.
