# Supplementary material for: Three Staphylococcus Bacteriophages Isolated from Swine Farm Environment in Quebec, Canada, Infecting S. chromogenes
Source: Viruses. 2026 Jan 22;18(1):146. doi: 10.3390/v18010146 (PMC12846477; doi:10.3390/v18010146)
Supplement: Supplementary file 1 [file viruses-18-00146-s001.zip › viruses-4071233-supplementary.pdf]

SUPPLEMENTARY MATERIALS

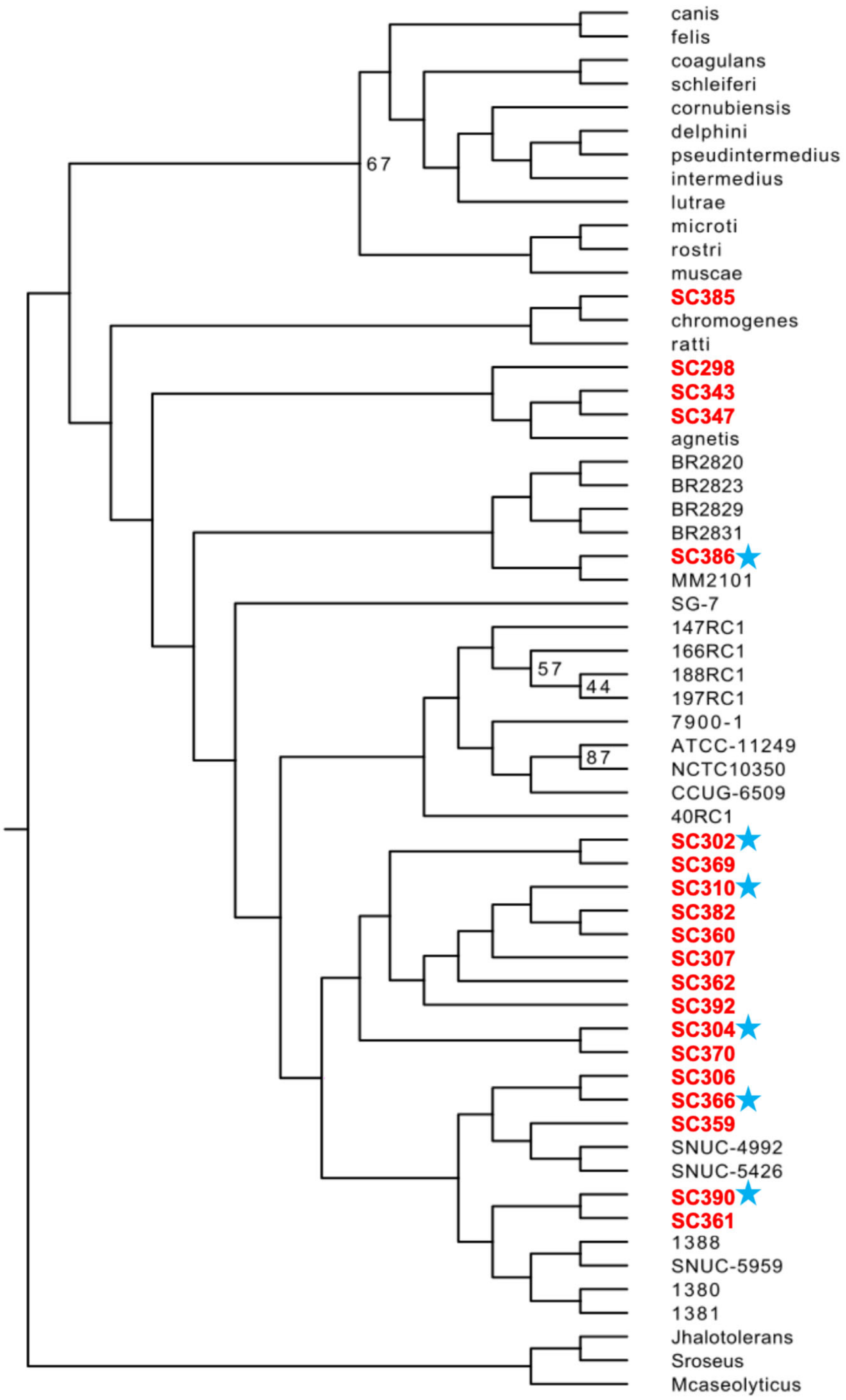

Figure S1. Phylogenetic tree of strains of the genus *Staphylococcus* focusing on taxon near *S. hyicus*, used as a reference to select host strains for environmental phage isolation. Strains in red are *Staphylococcus* strains available in our lab for which genomic sequence were available at the start of this project. Of those, 6 strains (SC302, SC304, SC310, SC366, SC386, SC390) with blue star marks were used as hosts in the initial environmental phage isolation of this project. Bootstrap

values are shown only when they are below 100, for clarity. The phylogenetic tree was constructed using a protocol published by Gagné-Thivierge et al. [3].

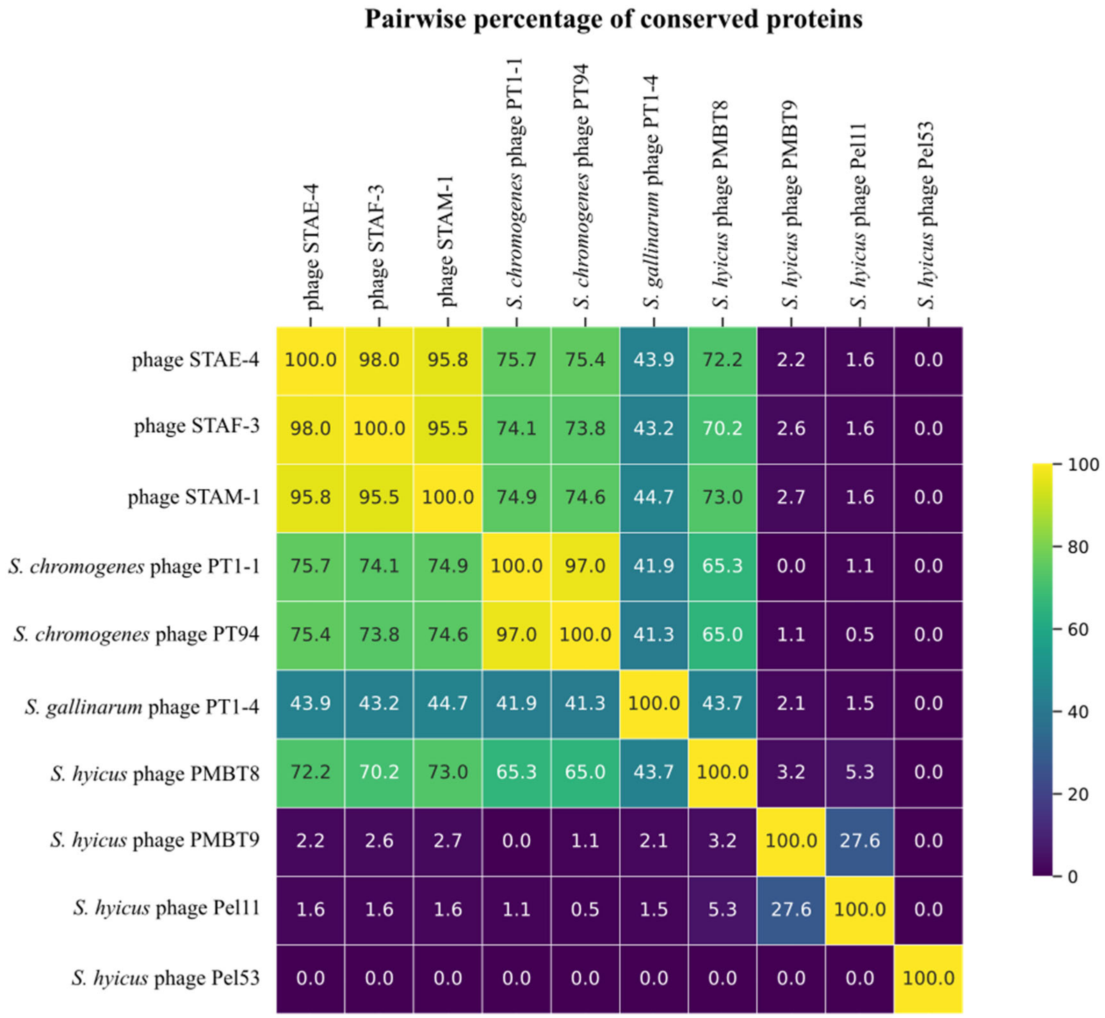

Figure S2. Heatmap representing the pairwise percentage of conserved proteins between all phages in this study. Percentage of conserved proteins values are indicated for each comparison and the conservation-level is represented by a color gradient: from low conservation (purple) to high conservation (yellow).

Table S1. Bacterial strains used in this study. Empty cells indicate that no information is available. a: ATCC : American Type Culture Collection (Manassas, USA); FHRC: Félix D'Hérelle Reference Center for Bacterial Viruses (Quebec, Canada); LSA: Laboratoire de santé animale, Ministère de l’Agriculture, des Pêcheries et de l’Alimentation du Québec (MAPAQ) (Saint-Hyacinthe, Canada); MPCC : Mastitis Pathogen Culture Collection (Saint-Hyacinthe, Canada).

Species	Name	Alternative name	Genome Assemblies	Sequence Reads	Source[a]	Refe-rences
S. hyicus	SC299	ATCC11249	GCA_000816085.1		ATCC	[1]
	SC300	84-2978			LSA	[2]
	SC302		GCA_030709895.1		LSA	[3]
	SC304		GCA_030709925.1		LSA	[3]
	SC306		GCA_030709835.1		LSA	[3]
	SC310		GCA_030709795.2		LSA	[3]
	SC303				LSA	
	SC305				LSA	
	SC307		GCA_042608445.1		LSA	
	SC308				LSA	
	SC309				LSA	
	SC311		GCA_042608535.1		LSA	
	SC357				LSA	
	SC358				LSA	
	SC359			SRX29800532	LSA	
	SC360		GCA_042608415.1		LSA	
	SC361			SRX29800533	LSA	
	SC362		GCA_042608545.1		LSA	
	SC363				LSA	
	SC364				LSA	
	SC365				LSA	
	SC366			SRX29800535	LSA	
	SC367				LSA	
	SC368		GCA_042608395.1		LSA	
	SC369			SRX29800536	LSA	
	SC370			SRX29800537	LSA	
	SC371				LSA	
	SC372				LSA	
	SC373		GCA_042608475.1		LSA	
	SC374		GCA_042608505.1		LSA	
	SC375				LSA	
	SC376				LSA	
	SC377				LSA	
	SC378				LSA	
	SC379				LSA	
	SC380				LSA	
	SC381				LSA	
	SC382		GCA_042608405.1		LSA	

	SC383				LSA	
	SC384				LSA	
	SC386			SRX29800538	LSA	
	SC387				LSA	
	SC388				LSA	
	SC389				LSA	
	SC390			SRX29800539	LSA	
	SC391				LSA	
	SC392		GCA_042608455.1		LSA	
	SC393		GCA_042608465.1		LSA	
	SC394				LSA	
S. agnetis	SC298	HER1048		SRX29800540	FHRC	
	SC341	10000719			MPCC	[4]
	SC342	20000012			MPCC	[4]
	SC343	20000033		SRX29800541	MPCC	[4]
	SC344	20000076			MPCC	[4]
	SC345	20000136			MPCC	[4]
	SC346	20000188			MPCC	[4]
	SC347	20000280		SRX29800542	MPCC	[4]
	SC348	20000563			MPCC	[4]
	SC349	20000662			MPCC	[4]
	SC350	20001262			MPCC	[4]
	SC351	20001537			MPCC	[4]
	SC352	20002039			MPCC	[4]
	SC353	20002180			MPCC	[4]
	SC354	20002230			MPCC	[4]
	SC355	20002429			MPCC	[4]
	SC356	20002995			MPCC	[4]
S. chromogenes	SC385			SRX29800534	LSA	
	SC426	11004936			MPCC	[4]
	SC427	22703910			MPCC	[4]
	SC428	21207662			MPCC	[4]
	SC429	11801702			MPCC	[4]
	SC430	10205785			MPCC	[4]
	SC431	10809372			MPCC	[4]
	SC432	11214502			MPCC	[4]
	SC433	30105348			MPCC	[4]
	SC434	10815496			MPCC	[4]
	SC435	31902335			MPCC	[4]
	SC436	41500149			MPCC	[4]
	SC437	40906768			MPCC	[4]
	SC438	40701059			MPCC	[4]

Table S2. Bacterial genomes used for the phylogenetic analysis presented in Figure S1.

**Species**	**Strain**	**Accession number**
Staphylococcus agnetis	1379	PRJNA553673
	SC298	PRJNA1294972
	SC343	PRJNA1294972
	SC347	PRJNA1294972
Staphylococcus canis	H16/1A	PRJNA601973
Staphylococcus coagulans	1031336	PRJNA734121
Staphylococcus cornubiensis	NW1	PRJEB20760
Staphylococcus chromogenes	22	PRJNA357351
	SC385	PRJNA1294972
Staphylococcus delphini	IVB6190	PRJNA819273
Staphylococcus felis	ATCC 49168	PRJNA438350
Staphylococcus hyicus	BR2831	PRJNA809943
	BR2829	PRJNA809943
	BR2823	PRJNA809943
	BR2820	PRJNA809943
	MM2101	PRJNA875208
	SG-7	PRJNA932936
	ATCC 11249	PRJNA252807
	SNUC 5426	PRJNA342349
	SNUC 4992	PRJNA342349
	SNUC 5959	PRJNA342349
	CCUG 6509	PRJNA339206
	1381	PRJNA553673
	1388	PRJNA553673
	1380	PRJNA553673
	janv-00	PRJNA609060
	166RC1	PRJNA862074
	40RC1	PRJNA862074
	197RC1	PRJNA862074
	188RC1	PRJNA862074
	147RC1	PRJNA862074
	NCTC10350	PRJEB6403
	SC302	PRJNA985240
	SC304	PRJNA985240
	SC306	PRJNA985240
	SC307	PRJNA985240
	SC310	PRJNA985240
	SC359	PRJNA1294972
	SC360	PRJNA985240
	SC361	PRJNA1294972
	SC362	PRJNA985240
	SC366	PRJNA1294972
	SC369	PRJNA1294972
	SC370	PRJNA1294972

	SC382	PRJNA985240
	SC386	PRJNA1294972
	SC390	PRJNA1294972
	SC392	PRJNA985240
Staphylococcus intermedius	NCTC 11048	PRJEB6403
Staphylococcus lutrae	ATCC 700373	PRJNA379140
Staphylococcus microti	NCTC13832	PRJEB6403
Staphylococcus muscae	NCTC13833	PRJEB6403
Staphylococcus pseudintermedius	SP_11304-3A	PRJNA683859
Staphylococcus ratti	CCM 9025	PRJNA779216
Staphylococcus rostri	DSM 21968	PRJNA339206
Staphylococcus schleiferi	MGYG-HGUT-01437	PRJEB33885
Jeotgalicoccus halotolerans	DSM 17274	PRJNA463390
Macrococcoides caseolyticum	FDAARGOS_868	SRS6867728
Salinicoccus roseus	W12	PRJNA272357

Table S3. Genomic characteristics of the three phages isolated in this study.

Phage	Genome size (kb)	GC conten t (%)	Predicted genes	Coding genes[a] (%)	Gene density[b] (genes per kb)	Putative function	Unknown function	Hypothetic al proteins
STAE-4	85.9	31.6	159	100	1.85	56	3	100
STAF-3	85.9	31.6	159	100	1.85	56	3	100
STAM-1	84.6	31.6	157	100	1.86	55	3	99

[a] Coding genes/predicted genes

[b] Predicted genes/genome size

Table S4. Average nucleotide identity (ANI) between the three phages isolated in this study and their closest known relatives.

	STAE-4	STAF-3	STAM-1	PT94	PT1-1	PMBT8	PT1-4
STAE-4	100	99.96	99.69	90.53	90.05	92.29	73.20
STAF-3		100	99.70	90.64	90.31	92.17	72.92
STAM-1			100	90.96	90.37	93.44	72.88
PT94				100	97.84	87.93	71.88
PT1-1					100	87.39	71.88
PMBT8						100	72.16
PT1-4							100

Table S5. Percentage of coverage between the three phages isolated in this study and their closest known relatives.

	STAE-4	STAF-3	STAM-1	PT94	PT1-1	PMBT8	PT1-4
STAE-4	100	98.78	95.22	56.85	56.66	52.55	19.40
STAF-3		100	96.07	54.18	56.17	55.74	18.97
STAM-1			100	54.99	55.24	53.99	19.95
PT94				100	91.53	43.88	17.18
PT1-1					100	42.43	15.95
PMBT8						100	17.65
PT1-4							100

Table S6. Number and functions of the coding sequences identified in the three phages isolated in this study and the previously described *S. chromogenes* phages PT1-1 and PT94.

Functions	STAE-4	STAF-3	STAM-1	PT1-1	PT94
Connector	4	4	4	4	4
DNA, RNA and nucleotide metabolism	24	24	22	24	22
Head and packaging	7	7	7	7	6
Integration and excision	1	1	1	0	0
Lysis	3	3	3	2	2
Moron, auxiliary metabolic gene and host takeover	2	2	2	3	3
Tail	4	4	4	4	4
Transcription regulation	1	1	1	1	1
Other	2	2	2	4	4
Unknown function	111	111	111	102	105
**Total CDS**	**159**	**159**	**157**	**151**	**151**

References

1. Calcutt, M.J., et al., *Sequence analysis of Staphylococcus hyicus ATCC 11249T, an etiological agent of exudative epidermitis in swine, reveals a type VII secretion system locus and a novel 116-kilobase genomic island harboring toxin-encoding genes.* Genome Announcements, 2015. **3**(1): p. e01525-14. https://doi-org.acces.bibl.ulaval.ca/10.1128/genomeA.01525-14
2. Vaillancourt, K., et al., *In vitro antibacterial activity of plant essential oils against Staphylococcus hyicus and Staphylococcus aureus, the causative agents of exudative epidermitis in pigs.* Archives of microbiology, 2018. **200**(7): p. 1001-1007. https://doi-org.acces.bibl.ulaval.ca/10.1007/s00203-018-1512-4
3. Gagné-Thivierge, C., et al., *Draft genome sequences of four Staphylococcus hyicus strains, SC302, SC304, SC306, and SC310, isolated from swine from Eastern Canada.* Microbiology Resource Announcements, 2023. **12**(10): p. e00626-23. https://doi.org/10.1128/MRA.00626-23
4. Dufour, S., J. Labrie, and M. Jacques, *The Mastitis Pathogens Culture Collection.* Microbiology Resource Announcements, 2019. **8**(15): p. 10.1128/mra.00133-19. https://doi.org/10.1128/mra.00133-19
